# A new cryptic species of *Chelidurella* Verhoeff, 1902 (Dermaptera, Forficulidae) from the Italian Alps: molecular evidence reveals hidden diversity in a high-altitude refugium

**DOI:** 10.3897/zookeys.1272.180451

**Published:** 2026-03-06

**Authors:** Petr Kočárek, Paolo Fontana, Ivona Kočárková, Vojtěch Bonczek

**Affiliations:** 1 Department of Biology and Ecology, Faculty of Science, University of Ostrava, Ostrava, Czech Republic Faculty of Science, University of Ostrava Ostrava Czech Republic; 2 Edmund Mach Foundation, San Michele all’Adige, Italy Edmund Mach Foundation San Michele all’Adige Italy; 3 World Biodiversity Association, c/o Museo Civico di Storia Naturale, Verona, Italy World Biodiversity Association, c/o Museo Civico di Storia Naturale Verona Italy

**Keywords:** Alpine biogeography, DNA barcoding, endemic species, Italy, Palaearctic region, phylogeny, taxonomy

## Abstract

The cryptic diversity within the earwig genus *Chelidurella* Verhoeff, 1902 remains underestimated despite growing evidence from molecular phylogenetic studies. During recent collecting efforts in the Adamello-Presanella Alps, a population of *Chelidurella* specimens that morphologically resemble *C. mutica* (Krauss, 1886) but exhibit distinct molecular divergence was discovered. Based on integrative taxonomic analysis combining molecular evidence with detailed morphological examination, *Chelidurella maccagnoae* Kočárek & Fontana, **sp. nov**. is described. Despite sharing the diagnostic shortened pygidium with *C. mutica*, phylogenetic analysis reveals that *C. maccagnoae* Kočárek & Fontana, **sp. nov**. is more closely related to the *C. vignai* / *C. pseudovignai* species complex, indicating convergent evolution of this character rather than shared ancestry. An updated identification key to *Chelidurella* males is provided and the biogeographic implications for understanding Quaternary diversification patterns in flightless Alpine arthropods discussed.

## Introduction

The genus *Chelidurella* Verhoeff, 1902 represents a complex of nine morphologically similar earwig species distributed across Europe. Majority of these flightless forficulids are restricted to high-altitude habitats, with most species showing narrow endemic distributions in the Alps (Galvagni 1997; Kirstová et al. 2021).

The taxonomic complexity of *Chelidurella* has long been recognised, with individual species traditionally delimited by subtle combinations of variable characters on the pygidium and forceps that often overlap between taxa (Capra 1982; Vigna Taglianti 1993; Galvagni 1994, 1995, 1996, 1997). This morphological similarity has led to considerable confusion in species identification and likely to the underestimation of true diversity within the genus. Recent molecular phylogenetic analyses have confirmed the existence of cryptic diversity, as demonstrated by the discovery of *C. pseudovignai* Kočárek & Kirstová, 2021, which is morphologically nearly identical to *C. vignai* Galvagni, 1995, but genetically well-differentiated and geographically separated (Kirstová et al. 2021).

The Alpine region has been recognised as a major centre of speciation and endemism for numerous arthropod groups, with Quaternary climatic oscillations promoting allopatric divergence in montane refugia (Schmitt 2009; Pauls et al. 2013). The restricted distributions and low dispersal ability of *Chelidurella* species make them particularly susceptible to such geographic isolation, potentially leading to cryptic speciation in isolated mountain massifs.

*Chelidurella mutica* (Krauss, 1886) is one of the most distinctive species in the genus, characterised by its small, broadly truncated pygidium (Kirstová et al. 2021). The species was described from the central-southern Alps of Italy (Monte Baldo), with some additional records from Austria and Switzerland (Galvagni 1997; Haas 2007). However, the true diversity within what has been considered “*C. mutica*” has never been thoroughly investigated using molecular tools.

During recent collecting efforts in the Italian Alps, we discovered a population of *Chelidurella* specimens from Passo Crocedomini that is morphologically very close to *C. mutica* but show distinct molecular divergence. Preliminary molecular analyses reveal that this population represents a cryptic species that, despite its morphological similarity to *C. mutica*, is phylogenetically closer to the species pair *C. pseudovignai* /*C. vignai*. This finding adds further evidence to the growing recognition of hidden diversity within *Chelidurella* and highlights the importance of integrative taxonomic approaches in revealing the true extent of Alpine biodiversity.

Here we describe this new cryptic species based on molecular phylogenetic analyses combined with detailed morphological examination, discuss its biogeographic implications for understanding Quaternary diversification in the Alps, and provide updated identification tools for the genus.

## Materials and methods

### Taxon sampling and processing

Specimens of describing species has been collected in Italy: Passo di Crocedomini, Brescia (BS), and, and preserved in 96% ethanol until tissue extraction for DNA isolation. For DNA isolation, we used leg and thoracic muscles. After that, the studied specimens were dissected and mounted on a label, with male genital armatures placed in small vial with glycerine and pinned to the card with the specimen. For the morphological comparisons, the specimens from the following localities were used: *Chelidurella mutica*: Italy: Veneto, Monte Baldo, 45°46'27.383"N, 10°52'5.160"E, 6.ix.2023, P. Kočárek leg.; *Chelidurella pseudovignai*: Italy: Carnia env., 550 m a.s.l., 46°23'06"N, 13°09'53"E, 20.ix.2015, sweeping of bushes, M. Kirstová leg.; *Chelidurella vignai*: Italy: Trento, 46°07'11"N, 11°15'40"E, loc. 7, 1031 m a.s.l., 12.ix.2015, M. Kirstová leg.

The specimens used for visualisation were photographed with a Leica Z16 APO macroscope (Leica Microsystems, Wetzlar, Germany) equipped with a CANON 6D Mark II camera. Micrographs of 10 to 20 focal layers of the same specimen were combined with Helicon Focus software and finally processed with Adobe Photoshop CS6 Extended (Version 13). The terminology used for morphological characteristics follows that of Steinmann (1986); the terminology used to describe the terminalia and genitalia follows that of Kamimura (2014). The nomenclature used follows that of Hopkins et al. (2025).

The material treated in this study is deposited in the following collections:

**FMCR** the Fondazione Museo Civico di Rovereto, Italy

**UO** the University of Ostrava, Department of Biology and Ecology, Czech Republic

### DNA extraction, amplification, and sequencing

Total genomic DNA was extracted from five Forficulidae taxa via the DNeasy Blood & Tissue Kit (QIAGEN, Inc.) following the manufacturer’s protocol. Partial sequences of three genetic markers (one nuclear and two mitochondrial) were amplified and sequenced. These include internal transcribed spacer 2 (ITS2), cytochrome c oxidase subunit I (COI), and 12S rRNA. Polymerase chain reactions (PCRs) were performed in 20-µl volumes containing 1 µl of DNA template, each primer at 0.4 µM, distilled water, and 1× PCRBIO HS Taq Mix Red (PCR Biosystems, London, UK). Primer pairs and PCR conditions used for amplification are listed in Table 1. The PCR products were visualised via 1% agarose gel electrophoresis with MIDORI Green Advance (NIPPON Genetics EUROPE) to confirm amplification. For purification of the amplified DNA was used a Gel/PCR DNA Fragments Extraction Kit (GENAID, Taiwan). Sanger sequencing reactions were performed at Eurofins Genomics (Konstanz, Germany). The chromatograms were visually checked via ChromasPro v2.1.10.1 software (Technelysium, Brisbane, Australia). Details of analysed taxa including isolation numbers, localities and GenBank accession numbers are indicated in Table 2.

**Table 1.** Primers and conditions used for PCR amplification.

Gene	Primer name	Primer sequence 5’**→**3’	PCR protocol	Primer reference
**COI**	jgLCO1490	TITCIACIAAYCAYAARGAYATTGG	2 min 30 s at 94 °C, [30 s at 90 °C, 1 min at 51 °C, 1 min at 72 °C] 40×, 10 min 30 s at 72 °C	Geller et al. 2013
	jgHCO2198	TAAACTTCAGGGTGACCAAAAAATCA		
**12S**	SR-N-14594	AAACTAGGATTAGATACCC	3 min at 95 °C, [30 s at 95 °C, 1 min at 52 °C, 1 min at 72 °C] 10×, [30 s at 95 °C, 1 min at 52 °C, 1 min at 72 °C] 25×, 7 min at 72 °C	Kambhampati and Smith 1995
	SR-J-14199	TACTATGTTACGACTTAT		
**ITS2**	CAS5p8sFc	TGAACATCGACATTTYGAACGCACAT	1 min at 94 °C, [15 s at 94 °C, 15 s at 53 °C, 15 s at 72 °C] 35×, 6 min at 72 °C	Ji et al. 2003
	CAS28sB1d	TTCTTTTCCTCCSCTTAYTRATATGCTTAA		

**Table 2.** Details of the specimens used in the phylogenetic analyses. Abbreviations: FMCR – the Fondazione Museo Civico di Rovereto, Italy; UO – University of Ostrava, Czech Republic; N/A – not available. New obtained sequences are marked in bold.

Analysed taxa	Locality and year of collection	Isolate / Voucher No. (deposition)	COI	12S	ITS2
**FORFICULIDAE Latreille, 1810**					
***Chelidura* Latreille, 1825**					
*Chelidura arverna* David & Van Herrewege, 1973	France: Chalmazel, 2016	C_pyr (OU)	MH853428	MH853366	MH853395
*Chelidura pyrenaica* (Bonelli, 1832)	Spain: Girona, 2011	230 (OU)	**PX585049**	**PX640174**	**PX640169**
*Chelidura transsilvanica* (Ebner, 1932)	Romania, Chișcău, 2022	392 (OU)	**PX585051**	**PX640175**	**PX640170**
*Chelidura* sp.	Italy: Lombardy (Sondrio), 2019	251 (OU)	**PX585048**	**PX640173**	**PX640168**
***Chelidurella* Verhoeff, 1902**					
*Chelidurella acanthopygia* (Gené, 1832)	Czech Republic: Ostrava, 2014	24CZ (OU)	MH853444	MH853378	MH853406
*Chelidurella fontanai* Galvagni, 1996	Italy: Genoa, 2015	091T (OU)	MH853435	MH853373	MH853401
*Chelidurella galvagnii* Kirstová & Kočárek, 2021	Austria: Karnten, 2015	01AU (OU)	MH853429	MH853367	N/A
*Chelidurella maccagnoae* Kočárek & Fontana, sp. nov.	Italy: Passo di Crocedomini, 2024	481 (FMCR)	**PX585052**	**PX640177**	**PX640172**
*Chelidurella mutica* (Krauss, 1886)	Italy: Veneto, Monte Baldo, 2023	444 (OU)	**PX585050**	**PX640176**	**PX640171**
*Chelidurella pseudovignai* Kočárek & Kirstová, 2021	Austria: Carinthia, 2015	04AU (OU)	MH853431	MH853369	MH853397
*Chelidurella thaleri* Harz, 1980	Italy: Bolzano, 2015	07IT (OU)	MH853434	MH853372	MH853400
*Chelidurella vignai* Galvagni, 1995	Italy: Trento, 2015	05IT (OU)	MH853430	MH853368	MH853396
**Outgroup**					
*Anechura bipunctata* (Fabricius, 1781)	Mongolia: Ikh-tamir, 2016	A_bip (OU)	MH853426	MH853364	N/A
*Mesochelidura occidentalis* de Fernandes, 1973	Portugal: Monchique, 2017	M_occ (OU)	MH853427	MH853365	N/A

### Phylogenetic analysis

The sequences were aligned using MAFFT with default parameters implemented via the EMBL-EBI sequence analysis tools (Madeira et al. 2022). Pairwise genetic distances using the Kimura 2-parameter model were calculated for the COI and 12S alignments in MEGA v12 (Kumar et al. 2024).

For phylogenetic reconstruction, we used partial sequences of three genetic markers: COI (657 bp), 12S rRNA (~617 bp), and ITS2 (~457 bp). The protein-coding gene (COI) was translated into amino acids to check for potential stop codons within the open reading frames. The multigene dataset of 1,731 bp was concatenated using Concatenator v0.2.1 (Vences et al. 2022). The best-fitting partitioning schemes and molecular evolution models were selected under the corrected Akaike information criterion via PartitionFinder v2.1.1 (Lanfear et al. 2017). Bayesian inference (BI) and maximum likelihood (ML) analyses were performed to infer phylogenetic relationships, and both analyses were run on the CIPRES Science Gateway v3.3 (Miller et al. 2010). Bayesian analysis was conducted with MrBayes v3.2.7a in XSEDE (Ronquist et al. 2012) via the Markov chain Monte Carlo (MCMC) method. Two independent MCMC runs of four chains were run for 5 million generations, and 25% of the trees were discarded as burn-in. The convergence of BI analysis was conﬁrmed in Tracer v1.7.2 (Rambaut et al. 2018). The maximum likelihood analysis based on the GTR+G+I nucleotide model was conducted via RAxML-HPC BlackBox v8.2.12 (Stamatakis 2014). The obtained trees were rooted by outgroup taxa (*Anechura bipunctata* and *Mesochelidura occidentalis*) and were displayed in iTOL (interactive Tree of Life) v6.8.1 (Letunic and Bork 2021).

## Results

### Taxonomy

#### Genus Chelidurella

Taxon classificationAnimaliaDermapteraForficulidae

Verhoeff, 1902

70333C86-3D42-5A69-B857-A0A4720AF51D

Chelidurella Verhoeff, 1902: 187. Type species: Forficula acanthopygia Gené, 1832 by subsequent designation in Kirby 1904: 43. Type locality: Alpes-Maritimes.

##### Included species.

*C. acanthopygia* (Gené, 1832); *C. caprai* Vigna Taglianti, 1993; *C. fontanai* Galvagni, 1996; *C. galvagnii* Kirstová & Kočárek, 2021; *C. mutica* (Krauss, 1886); *C. poggii* Capra, 1982; *C. pseudovignai* Kočárek & Kirstová, 2021; *C. thaleri* Harz, 1980; *C. vignai* Galvagni, 1995; *C. maccagnoae* Kočárek & Fontana, sp. nov.

##### Distribution.

Europe.

#### Chelidurella maccagnoae

Taxon classificationAnimaliaDermapteraForficulidae

Kočárek & Fontana sp. nov.

4A6995FB-A40F-5EEE-9188-958EEEA03693

https://zoobank.org/1DAB9F3B-5ED2-47E0-B989-5F3B6B2F146A

Figs 1, 2, 3

##### Material.

***Holotype***. Italy • 1 ♂; Passo di Crocedomini (Breno) BS, 1900 m; 45°54'19"N, 10°24'33"E; 19 Aug. 2024; Paolo Fontana leg.; GenBank: COI: PX585052, 12S: PX640177, ITS2: PX640172 (the Fontana collection in FMCR). ***Paratypes***. Italy • 1 ♀; Passo di Crocedomini (Breno) BS, 1900 m; 45°54'19"N, 10°24'33"E; 19 Aug. 2024; Paolo Fontana leg. (the Fontana collection in FMCR); Italy • 2 ♀; Passo di Crocedomini (Breno) BS, 1900 m; 45°54'28.7"N, 10°24'33.6"E, 5 Sep. 2023, Paolo and Carlotta Fontana leg.; ID: 454, 455 (UO).

##### Diagnosis.

*Chelidurella maccagnoae* Kočárek & Fontana, sp. nov. and *C. mutica* share shortened pygidium without upcurved projection and this character clearly separates these two species from the remaining described species of *Chelidurella*. *Chelidurella maccagnoae* sp. nov. and *C. mutica* are externally indistinguishable, but differ in the morphology of male genitalia. The penis lobe of *C. maccagnoae* sp. nov. is long, reaches almost the tip of the parameres, is nearly parallel sided and truncated distally; the penis lobe of *C. mutica* is shorter, reaches up to two-thirds of the length of parameres, sloping to the apex that is truncate. The parameres of *C. maccagnoae* sp. nov. are more angular along the outer margin than those of *C. mutica*. Both species differ also in the morphology of basal vesicle, in the shape of proximal expansion. Proximal and distal corners of the proximal expansion of basal vesicle are pointed in *C. maccagnoae* sp. nov., but broadly rounded in *C. mutica*.

##### Description.

**Figure 1.**
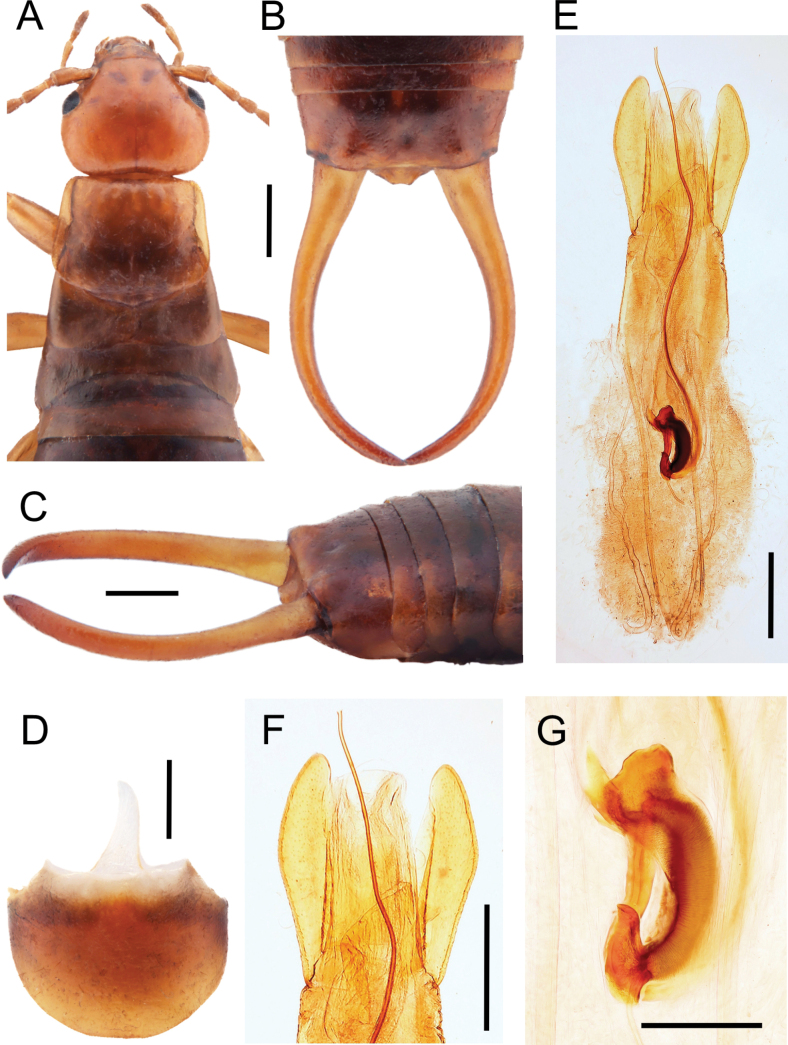
*Chelidurella maccagnoae* Kočárek & Fontana, sp. nov., habitus of holotype male. **A**. Dorsal view to head and thoracal segments; **B**. Ultimate tergite and cerci from dorsal view; **C**. Ultimate tergite and cerci from lateral view; **D**. Penultimate sternite, ventral view; **E**. Dorsal view on the genital of holotype; **F**. Detail of the tip of genital; **G**. Basal vesicle, ventral view. Scale bars: 1.0 mm (**A–D**); 0.5 mm (**E, F**); 0.2 mm (**G**).

**Figure 2.**
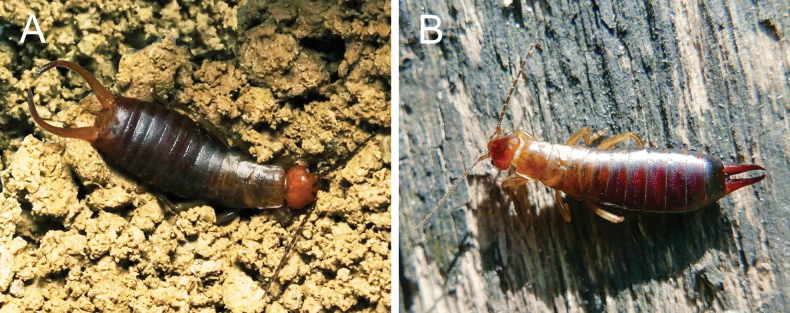
*Chelidurella maccagnoae* Kočárek & Fontana, sp. nov., live habitus of holotype male (**A**) and paratype female (**B**). Passo di Crocedomini (Breno), 1900 m. Photograph: PF 19 Aug. 2024.

Body yellowish-brown (Figs 1, 2); head orange-yellow, legs and forceps ochre. Cuticle punctured, shiny. Tegmina of *Chelidurella* type, rudimentary, rugose, with a short section overlapping medially; wings entirely absent. Pygidium ochre, short, without upward projection. Measurements of holotype male: Total length without forceps 11.8 mm, length of forceps 4.6 mm. Maximal head width 2.18 mm, maximal head length 2.1 mm, maximal pronotum length/width 1.7/2.2 mm, maximal tegmina length 2.49 mm, length of 1^st^ antennomere (scapus) 0.7 mm, length of 2^nd^ antennomere (pedicel) 0.1 mm, length of 3^rd^ antennomere 0.4 mm. Total length of male genitalia 3.2 mm (with exclusion of exceeding virga), basal vesicle length 0.4 mm. Measurements of paratype female: Total length without forceps 11.3 mm, length of forceps 2.3 mm in females.

**Holotype male. *Head*** (Fig. 1A) transverse; frons gibbose convex, postfrontal and coronal sutures distinct, posterior margin concave in middle. Eyes small, as long as 3^rd^ antennomere. Antennae with 13 antennomeres; 1^st^ antennomere narrowed in basal third, widened distally, as long as 3^rd^ and 4^th^ antennomeres combined; 2^nd^ antennomere short, quadratic; 3^rd^ antennomere longer than 4^th^, both subconical; antennomeres 5–13 cylindrical, narrowed at extreme base. All antennomeres shortly pubescent.

***Pronotum*** (Fig. 1A) transverse, as wide as head, slightly expanded distally and broadly rounded posteriorly. Disc of pronotum flat, median sulcus fine but distinct. Tegmina rugose, rudimentary, with a short section overlapping medially, lateral and internal edges broadly rounded. Mesonotum dorsally visible as distally broadly rounded mesoscutellum; metanotum transverse, wider than long, posteriorly broadly emarginate. Sternal plates typical for genus. Legs unicolourous ochre; femora stout; tibiae clad with thick and fine setae; 1^st^ metatarsomere cylindrical, longer than 2^nd^ and 3^rd^ tarsomeres combined, pulvilli not developed. Claws simple, symmetrical; arolium absent.

***Abdomen*** (Fig. 1B, C) somewhat widened in middle, lateral glandular folds present on tergites 3 and 4, tergites 4–6 rugose-striate at sides. Ultimate tergite transverse, slightly sloping posteriorly, more than twice as wide as long, median part somewhat depressed near posterior margin, disc with a pair of rounded tubercles near the forceps bases. Ninth sternite (penultimate) broadly rounded posteriorly (Fig. 1D). Pygidium broad and short, without upward projection, lateral sides sloping to central truncated part with small tubercules at corners. Forceps (Fig. 1B, C) arcuate, without any tooth, branches cylindrical and regularly tapering to the apex.

**Figure 3.**
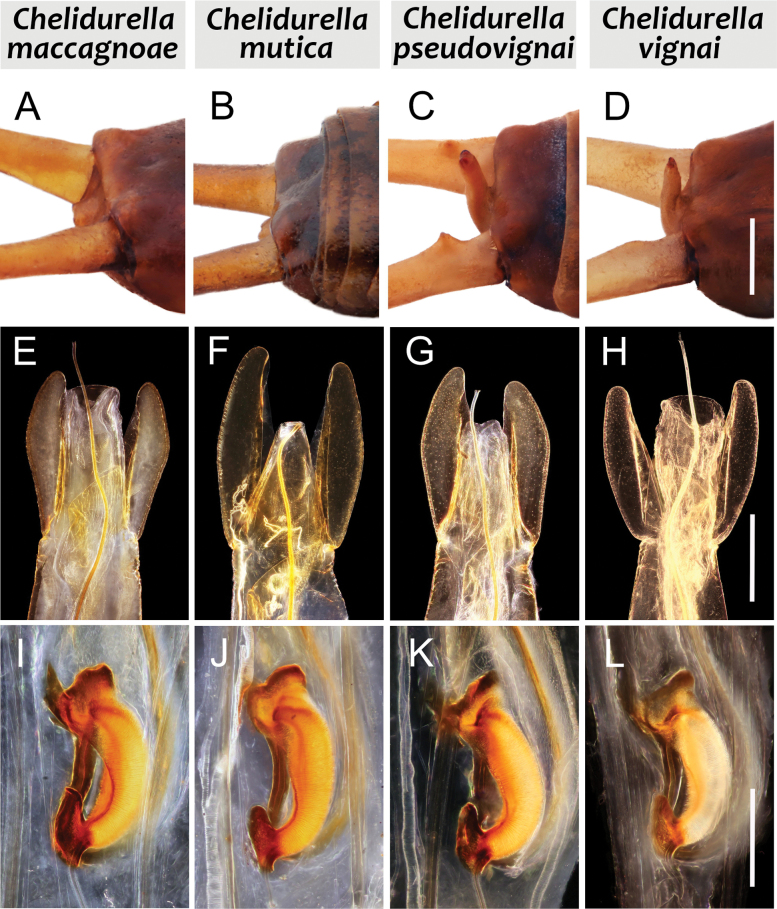
Comparison of male pygidiums, penis lobes, parameres, and basal vesicles. **A, E, I**. *Chelidurella maccagnoae* Kočárek & Fontana, sp. nov.; **B, F, J**. *C. mutica* (Krauss, 1886); **C, G, K**. *C. pseudovignai* Kočárek & Kirstová, 2021; **D, H, L**. *C. vignai* Galvagni, 1995. Scale bars: 1.0 mm (**A–D**); 0.5 mm (**E–H**); 0.2 mm (**I–L**).

***Genitalia*** (Figs 1E–G, 3E, 3I) with only slightly narrowed penis lobe distally with truncated apex. Parameres thin, 4× longer than wide, broadened in middle and narrowed apically, external margin convex, internal margin gently emarginated. Basal vesicle thin, 2× shorter than length of paramere, reniform. Proximal expansion more robust than distal expansion, distal expansion transverse, with acute corners. Virga sinuated, well sclerotised, elongated ~ 3× longer than paramere.

**Female**. Agrees with male in all characters except female forceps have simple and straight with contiguous branches (Fig. 2B).

##### Molecular identification.

We obtained partial COI sequence (657 bp) from the holotype male specimen of *Chelidurella maccagnoae* Kočárek & Fontana, sp. nov. as DNA barcode for molecular identification of the species. The barcode is deposited in GenBank under accession number PX585052 (DNA isolate number: 481).

##### Etymology.

The new species is dedicated to the Italian zoologist Teresita Maccagno Paulucci (1900–1999), who, with her seminal works, made a decisive contribution to the study of Dermaptera by pioneering the comparative examination of male genitalia in the classification of these insects (Maccagno 1926, 1933). Her life spanned a century, but, like many women in science, she never received the recognition she deserved.

##### Biology.

The few specimens collected (during intense searches) were identified under stones of various sizes at the edges of pastures near the Cricedomini Pass. Specimens were collected (in very small numbers) between the end of August (2024) and the beginning of September (2023). The species was also the subject of research in mid-July 2025, but without success. During the same field trip conducted at the end of August 2024, some juvenile stages of Dermaptera were identified in addition to the adult male and female collected, apparently attributable to the new species. This very limited data does not allow us to outline the phenology of this species, which appears to be present in a very small population.

##### Distribution.

Known only from type locality in Adamello-Presanella Alps in Italy, the Crocedòmini Pass. This is an Alpine pass in the Brescia Prealps, located in the province of Brescia at 1,892 m a.s.l., south of the Adamello Park in Lombardy. From an orographic perspective, the pass separates the Southern Rhaetian Alps from the Brescian and Garda Prealps. It is located just north of the point where the Three Brescian Valleys meet: Val Trompia, Val Camonica, and Val Sabbia.

### Key to males of *Chelidurella* (modified from Kirstová et al. 2021)

**Table:** 

1	Pygidium small, rounded. Viewed from the back, it does not reach the top line of the 10^th^ tergite (Fig. 3A, B)	**2**
–	Pygidium triangular, pointed at the end or truncated, and when viewed from the back, pygidium reaches or exceeds the upper line of the 10^th^ tergite (Fig. 3C, D)	**3**
2	Penis lobe nearly as long as parameres, robust, only slightly tapering towards the top (Fig. 3E). Proximal expansion of basal vesicle pointed (Fig. 3I)	***C. maccagnoae* sp. nov**.
–	Penis lobe narrower, conspicuously trapezoidal, reaching maximally to 2/3 of parameres (Fig. 3F). Proximal expansion of basal vesicle rounded (Fig. 3J)	***C. mutica* (Krauss, 1886)**
3	Pygidium long, elongated; viewed from the back, pygidium extends over the upper edge of the 10^th^ tergite ≤ 0.3× the body thickness measured from pygidium base to the upper line of the 10^th^ tergite	**4**
–	Pygidium short, trigonal; viewed from the back, pygidium either does not reach the upper edge of the 10^th^ tergite, or it extends only to 0.3× the body thickness measured from pygidium base to the upper line of the 10^th^ tergite	**6**
4	Pygidium very long; viewed from the back, pygidium extends over the upper edge of the 10^th^ tergite ≤ 2× the body height measured from pygidium base to this line. Distal 2/3 of pygidium narrow with parallel margins; pygidium is not strongly S-shaped viewed from the side	***C. fontanai* Galvagni, 1996**
–	Pygidium long, viewed from the back, pygidium extends over the upper edge of the 10^th^ tergite 0.3–0.8× the body height measured from pygidium base to this line. Distal 2/3 of pygidium narrow with convergent margins; pygidium is typically S-shaped viewed from the side	**5**
5	Parameres broadly rounded externally, not angulated in distal third. Basal vesicle as in Fig. 3L (molecular identification is recommended)	***C. vignai* Galvagni, 1995**
–	Parameres slightly but clearly angled externally in distal 1/3. Basal vesicle as in Fig. 3K (molecular identification is recommended)	***C. pseudovignai* Kočárek & Kirstová, 2021**
6	Antennomeres short and robust. Length/width ratio of antennomere 4 is maximally 1.5	**7**
–	Antennomeres long and thin. Length/width ratio of the antennomere 4 is ≤ 1.6	**8**
7	Pygidium with shape of an isosceles triangle; viewed from the back, pygidium barely reaches the upper edge of the 10^th^ tergite, or it extends over the edge maximally 0.25× the body thickness measured from the pygidium base to the upper edge of tergite 10	***C. poggii* Capra, 1982**
–	Basal part of pygidium triangular; distal part with the narrow projection with nearly parallel sides. Apex of pygidium truncated with two protuberances	***C. galvagnii* Kirstová & Kočárek, 2021**
8	Subgenital plate with trigonally tapering distal 1/3	***C. caprai* Vigna Taglianti, 1993**
–	Subgenital plate nearly parallel-sided on proximal 2/3, with rounded sides on distal 1/3	**9**
9	Penis lobe relatively long; paramere ≤ 2× longer than maximal width of pronotum; distal width of penis lobe ≤ 2× broader than maximal width of paramere	***C. thaleri* Harz, 1980**
–	Penis lobe relatively short; paramere maximally 1.6× longer than maximal width of pronotum; distal width of penis lobe approximately as broad as maximal width of paramere	***C. acanthopygia* (Gené, 1832)**

### Phylogenetic analyses

**Figure 4.**
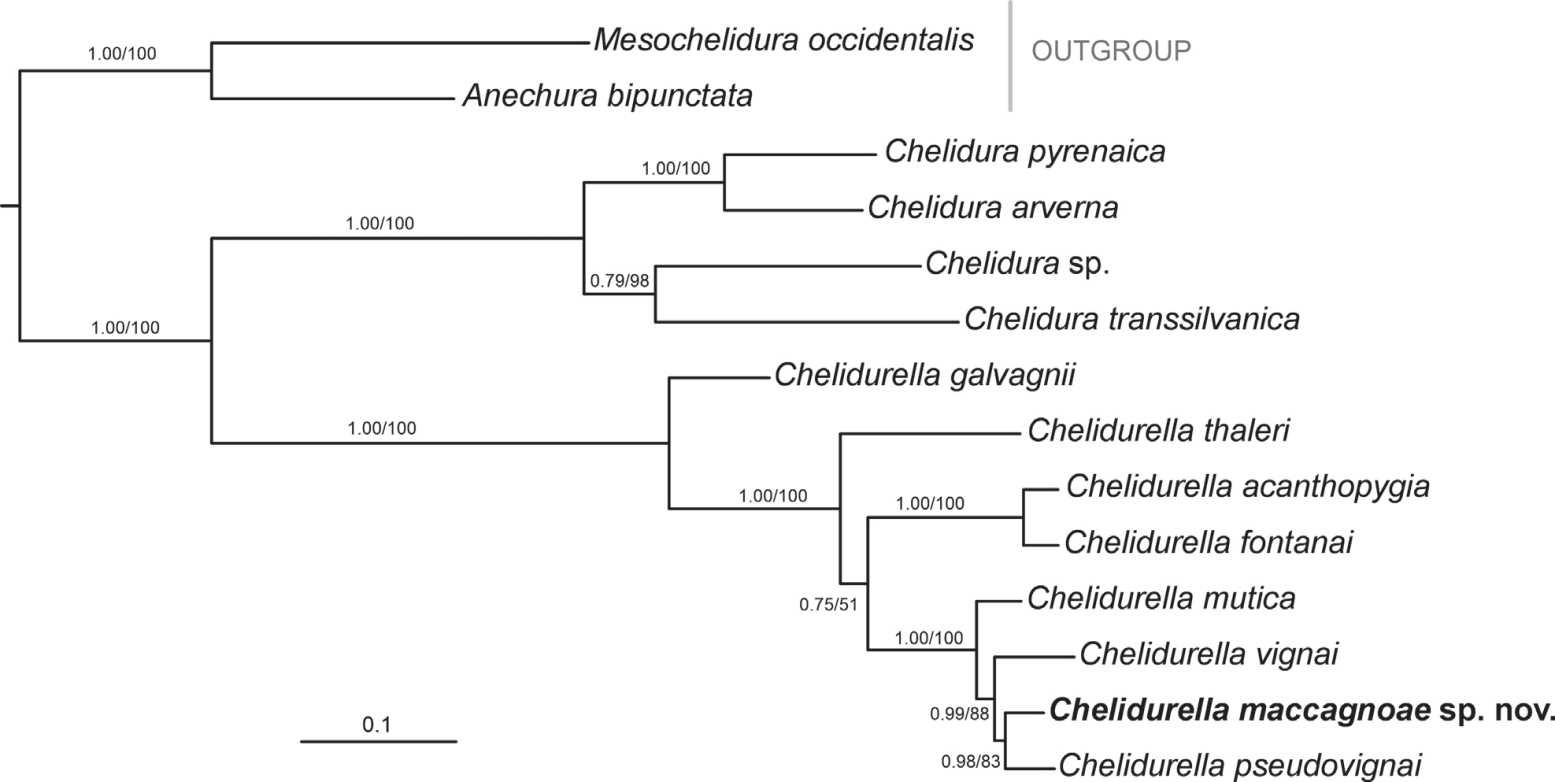
Phylogenetic tree resolved by Bayesian inference based on the combined dataset for three molecular markers (COI, 12S rRNA, and ITS2). Bayesian posterior probabilities (PP) and RAxML bootstrap support (BS) values are given at each node in the following order: PP/BS.

The analyses contain eight species of the genus *Chelidurella*, including the new species described in this study, and four species of the genus *Chelidura* Latreille, 1825. The dataset of three molecular markers (COI, 12S, and ITS2) comprised 1,731 bp. The trees based separately on the BI and ML methods produced identical topologies (Fig. 4). The genus *Chelidurella* forms a monophyletic clade with 100% support in both analyses in a sister position to the genus *Chelidura*.

*Chelidurella mutica*, *C. vignai*, *C. pseudovignai*, and *C. maccagnoae* sp. nov. form a monophyletic clade that is sister to the clade comprising *C. acanthopygia* and *C. fontanai*. The new species, *C. maccagnoae*, is the sister species to *C. pseudovignai*. The pairwise genetic distances (Kimura 2-parameter model) between these two species were 6.7% for COI and 2.8% for 12S. The pairwise genetic distances for all analysed *Chelidurella* species (COI and 12S) are presented in Table 3.

**Table 3.** Sequence divergence of *Chelidurella* species for COI (657 bp) and 12S rRNA (424 bp). The values of COI are below the diagonal.

	Species	1	2	3	4	5	6	7	8
**1**	*Chelidurella acanthopygia*		0.031	0.169	0.119	0.128	0.122	0.128	0.146
**2**	*Chelidurella fontanai*	0.051		0.161	0.113	0.122	0.114	0.131	0.137
**3**	*Chelidurella galvagnii*	0.203	0.215		0.122	0.128	0.126	0.136	0.155
**4**	*Chelidurella maccagnoae* sp. nov.	0.181	0.189	0.183		0.028	0.028	0.115	0.071
**5**	*Chelidurella mutica*	0.190	0.193	0.176	0.086		0.039	0.127	0.082
**6**	*Chelidurella pseudovignai*	0.186	0.200	0.167	0.067	0.086		0.122	0.063
**7**	*Chelidurella thaleri*	0.174	0.165	0.183	0.180	0.164	0.183		0.139
**8**	*Chelidurella vignai*	0.178	0.180	0.182	0.074	0.080	0.071	0.174	

## Discussion

The cryptic diversity of the genus *Chelidurella* has long been recognised, but small morphological differences and high intraspecific variability have complicated species identification (Kirstová et al. 2021). While Italian entomologists Capra, Vigna Taglianti, and Galvagni made significant taxonomic contributions (Capra 1982; Vigna Taglianti 1993; Galvagni 1994, 1995, 1996, 1997), recent molecular revisions not only confirmed most described species but revealed additional cryptic taxa like *C. pseudovignai* (Kirstová et al. 2021). The discovery of *C. maccagnoae* sp. nov. through molecular verification of an isolated *C. mutica* population exemplifies this hidden diversity. Surprisingly, despite sharing an extremely shortened pygidium with *C. mutica*, *C. maccagnoae* sp. nov. is phylogenetically closer to the *C. vignai*/*C. pseudovignai* complex, indicating convergent evolution (homoplasy) rather than shared ancestry. This pygidial shortening likely represents adaptation to high-altitude conditions, potentially following Allen’s rule, though in ectothermic insects such morphological variation may reflect allometric growth under different climatic conditions rather than thermoregulation (Bidau and Martí 2008; Shelomi and Zeuss 2017).

Quaternary climatic oscillations have profoundly shaped Alpine invertebrate communities, particularly affecting flightless and brachypterous insects (Ikeda et al. 2012; Hunt et al. 2013). While classical European phylogeography emphasised three Mediterranean refugia (Iberian, Italian, Balkan) with northward postglacial expansion (Taberlet et al. 1998; Hewitt 2000), molecular studies reveal more complex patterns including peripheral refugia, nunatak refugia within mountain ranges (ice-free rocky peaks), and extra-Mediterranean refugia (Schönswetter et al. 2005; Schönswetter and Tribsch 2019; Stewart and Lister 2019). Flightless arthropods demonstrate pronounced Quaternary diversification effects due to limited dispersal. Studies of ground beetles reveal strong genetic differentiation with divergence times extending 1.6–2.5 million years (Raupach et al. 2013), while flightless species retain twice the speciation rates of flying relatives (Ikeda et al. 2012). The endemic bush-cricket *Anonconotus italoaustriacus* exemplifies Alpine flightless arthropod dynamics, with genomic evidence supporting survival in multiple peripheral refugia and subsequent southeastern recolonisation (Schönenberger et al. 2022).

The earwig genera *Chelidura* and *Chelidurella* exemplify contrasting evolutionary responses to Quaternary diversification. Both are completely flightless with rudimentary tegmina, severely limiting dispersal (Fontana et al. 2021; Kirstová et al. 2021). However, while *Chelidurella* species inhabit diverse environments from lowlands to upper forest boundaries, *Chelidura* species are exclusively high-altitude specialists (1400–2300 m), persisting above the tree line in harsh alpine conditions. Recent genetic analysis of *Chelidura aptera* species complex revealed substantial cryptic diversity among Alpine populations indicating long-term isolation in multiple refugia and significant taxonomic underestimation (Fontana et al. 2021). Their ecological requirements for alpine grasslands with rocky outcrops align perfectly with nunatak refugia scenarios, where ice-free peaks provided suitable refugial environments during Pleistocene glaciations. This ecological contrast drives different diversification patterns. *Chelidurella* shows four strongly supported molecular clades with probable Pleistocene diversification, yet even this ecologically flexible genus exhibits cryptic species pairs like *C. vignai* and *C. pseudovignai* in alpine environments (Kirstová et al. 2021). Notably, only high-altitude specialists *C. mutica* and the newly described species approach *Chelidura’s* ecological niche at ~2000 m elevation.

Both *Chelidura* and *Chelidurella* diversity patterns align with broader phylogeographic paradigms. Their restriction to high-altitude habitats, flightless condition, and specialised ecological requirements have promoted allopatric speciation within isolated mountain refugia. Molecular phylogenies indicate relatively recent, likely Pleistocene diversification, consistent with other Alpine endemic lineages showing Quaternary rather than Tertiary origins (Kadereit et al. 2004; Schmitt 2007).

These phylogeographic patterns have significant conservation implications under ongoing climate change. Historical Alpine refugia are becoming increasingly important as potential climate refugia for cold-adapted species, yet current protected area networks may inadequately cover these future refugia (Wilkes et al. 2022, 2023). High-altitude specialists like *Chelidura* species face particular vulnerability, as their narrow ecological tolerances and restricted distributions limit upward range shift capacity in response to warming temperatures.

## Supplementary Material

XML Treatment for Chelidurella

XML Treatment for Chelidurella maccagnoae

## Additional information

### Use of AI

No use of AI was reported.

### Author ORCIDs

Petr Kočárek https://orcid.org/0000-0002-1739-0143

Paolo Fontana https://orcid.org/0000-0001-9710-759X

Ivona Kočárková https://orcid.org/0000-0002-8942-9481

Vojtěch Bonczek https://orcid.org/0009-0004-8474-0096

### Data availability

All of the data that support the findings of this study are available in the main text.
